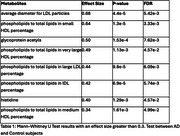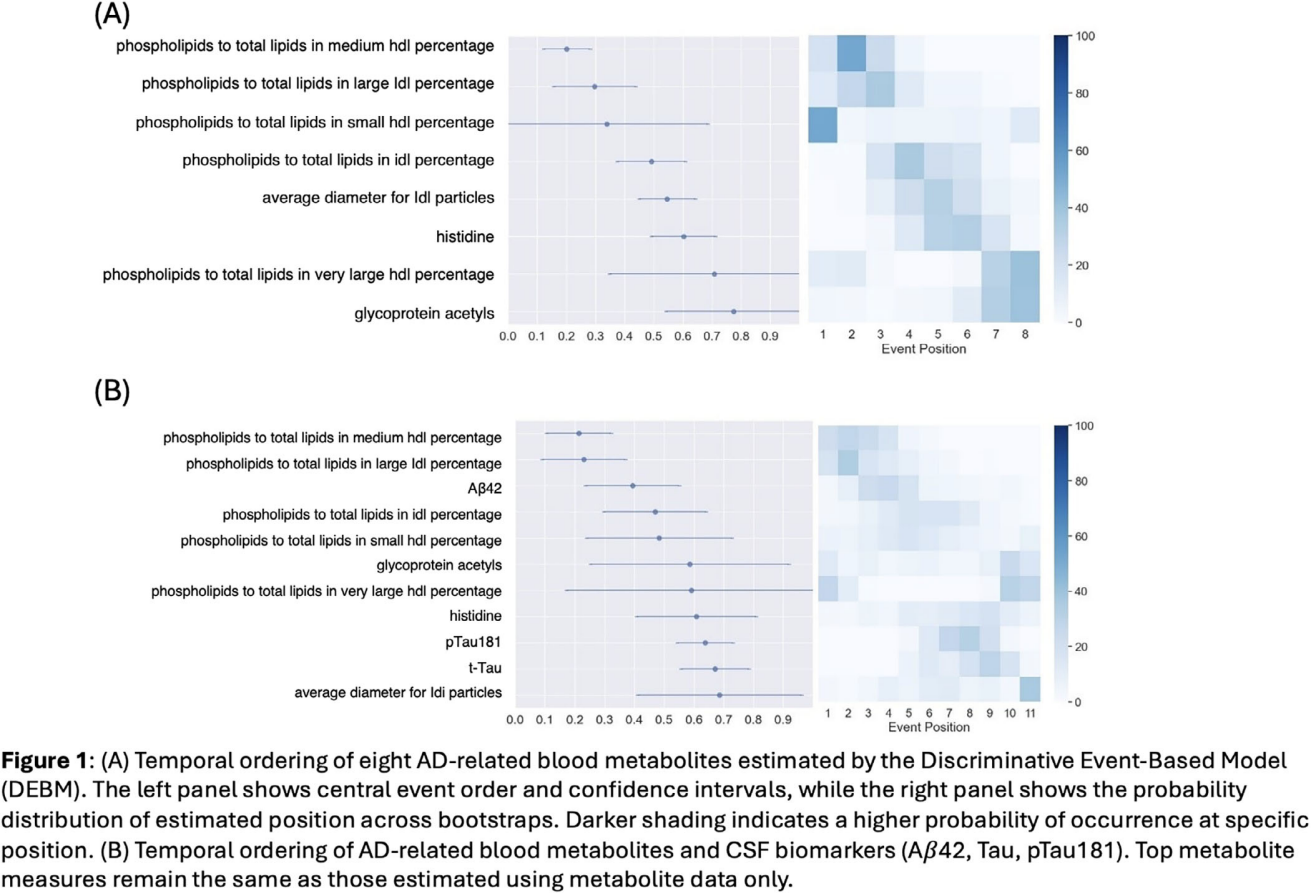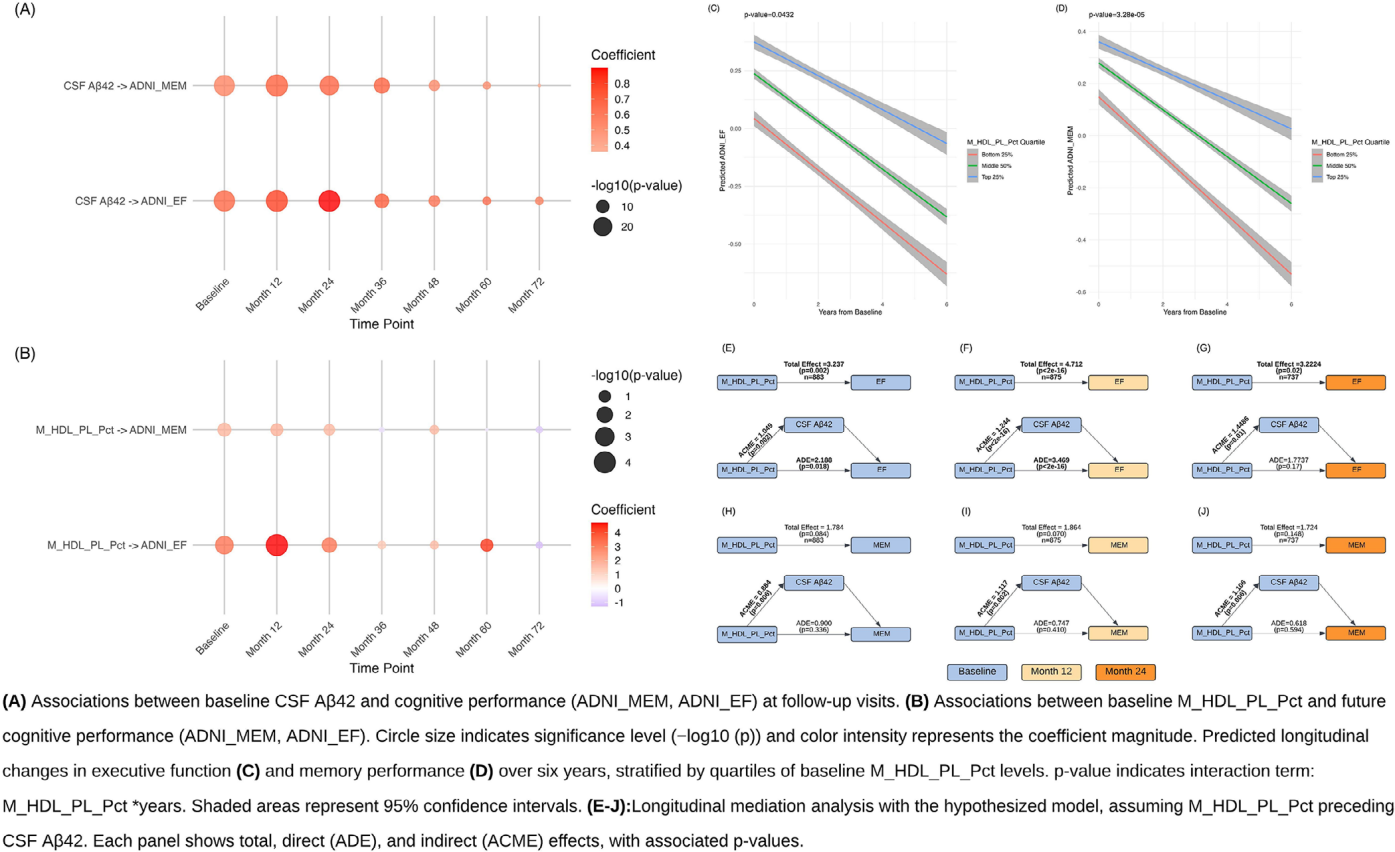# Temporal ordering of blood metabolites and CSF biomarkers reveals early changes in lipid composition in Alzheimer's disease

**DOI:** 10.1002/alz70856_104093

**Published:** 2025-12-25

**Authors:** Tianchuan Gao, Pradeep Varathan, Yen‐Ning Huang, Meichen Yu, Matthias Arnold, Rima F. Kaddurah‐Daouk, Andrew J. Saykin, Kwangsik Nho, Jingwen Yan

**Affiliations:** ^1^ Indiana university Indianapolis, Indianapolis, IN, USA; ^2^ Center for Neuroimaging, Department of Radiology and Imaging Sciences, Indiana University School of Medicine, Indianapolis, IN, USA; ^3^ Indiana Alzheimer's Disease Research Center, Indianapolis, IN, USA; ^4^ Indiana Alzheimer's Disease Research Center, Indiana University School of Medicine, Indianapolis, IN, USA; ^5^ Indiana University School of Medicine, Indianapolis, IN, USA; ^6^ Indiana University Network Science Institute, Bloomington, IN, USA; ^7^ Indiana Alzheimer's Disease Research Center, Indiana University School of Medicine, Indianapolis, IN, USA; ^8^ German Research Center for Environmental Health, Neuherberg, Neuherberg, Germany; ^9^ Department of Medicine, Duke University, Durham, NC, USA; ^10^ Duke University Medical Center, Durham, NC, USA; ^11^ Department of Psychiatry and Behavioral Sciences, Duke University, Durham, NC, USA; ^12^ Center for Neuroimaging, Indiana University School of Medicine, Indianapolis, IN, USA; ^13^ Indiana University School of Medicine, Department of Radiology and Imaging Sciences, Indianapolis, IN, USA; ^14^ Department of Radiology and Imaging Sciences, Indiana University School of Medicine, Indianapolis, IN, USA; ^15^ Center for Neuroimaging, Department of Radiology and Imaging Sciences, Indiana University School of Medicine, Indianapolis, IN, USA; ^16^ Indiana University Luddy School of Informatics, Computing and Engineering, Indianapolis, IN, USA

## Abstract

**Background:**

Although cerebrospinal fluid (CSF) biomarkers detect Alzheimer's disease (AD) changes early, their invasiveness and cost limit widespread use. Blood‐based metabolite measures, reflecting real‐time physiological states, may offer a non‐invasive alternative for early AD detection and monitoring.

**Method:**

Using data from the Alzheimer's Disease Neuroimaging Initiative, we measured 249 plasma metabolites via NMR spectroscopy and identified those significantly altered in AD compared to controls. We then applied Discriminative Event‐Based Modeling (DEBM) to estimate the temporal sequence of AD‐related blood metabolites alongside CSF biomarkers (Aβ42, total tau, phosphorylated tau). To validate these findings, we conducted longitudinal analyses assessing cross‐correlation with cognitive performance, mixed‐effects models relating baseline metabolites to changes in cognition over 72 months, and longitudinal mediation analyses to examine causal pathways.

**Result:**

Eight plasma metabolites were significantly altered in AD (Tab. 1). DEBM indicated that two metabolite ratios—phospholipids to total lipids in medium HDL and in large LDL—potentially preceded or coincided with CSF Aβ42 (Figure 1). Cross‐correlation analyses showed both baseline CSF Aβ42 and these metabolite ratios were most strongly associated with executive function and memory at 24 months (Figure 2). Linear mixed models revealed that higher phospholipids to total lipids ratio in medium HDL was linked to better cognition and slower cognitive decline. Longitudinal mediation analysis further supported a pathway where the medium HDL ratio preceded CSF Aβ42, which in turn influenced cognitive decline.

**Conclusion:**

Our results suggest that specific HDL lipid composition changes may occur very early in the AD cascade, slightly before or concurrent with CSF Aβ42 alterations. These findings support the potential utility of blood metabolite measures, particularly the phospholipids to total lipids ratio in medium HDL, as early AD biomarkers. Further research and replication in independent cohorts are needed to confirm these observations and advance non‐invasive AD screening.